# Preparing Learners for Learning in the Engaged Learning Classroom

**DOI:** 10.15694/mep.2019.000120.1

**Published:** 2019-05-31

**Authors:** Maryam Alizadeh, Dean Parmelee, Irina Overman, Mohamad AlJasem

**Affiliations:** 1 Tehran University of Medical Sciences; 2 Wright State University Boonshoft School of Medicine

**Keywords:** active learning, engaged classroom learning, flipped-classroom, engaged learning, Gen Z and learning in health sciences.

## Abstract

This article was migrated. The article was marked as recommended.

This set of Tips is written to make the best use of an Engaged Learning Classroom (ELC), which some may refer to as the ‘flipped classroom.’ Strategies for the ELC include Team-Based Learning (TBL), Peer Instruction (PI), Case-Based Learning (CBL). Our focus will be on the design and implementation of the out-of-class phase for any ELC activity since this is as important as the in-class phase, but often neglected. Experience has shown that the quality of learning from the ELC is highly dependent on the preparation before, and follow-up to the class session. The instructor’s designation of material (written, audio-visual, other potential learning resources and activities) and guidance on how to approach these materials generates engagement and enhances learning. We provide important contextual information on the learners of today (Generation Z), highlights of current learning theory applicable to self-directed learning, and the unique ingredients for the linkage between out-of-class and in-class learning that makes for the fullest student engagement. Each Tip addresses frequent questions by both instructors and learners who are embarking on the ELC and/or lessons that we have learned from the literature and designing, facilitating, and evaluating sessions.

## Introduction

Over the past decade, many undergraduate medical and health science education programs became learner-centered. The process began in the early 1990’s with the development of Problem-Based Learning (PBL), however, it was not until around 2007 that medical educators began to use TBL and CBL since these require fewer classrooms and faculty than PBL, and their learning activities also address professional competencies such as interpersonal communication and teamwork. In the U.S., the publication of the Carnegie Foundation’s report on medical education ( Irby, Cooke and O’Brien, 2010) for the 21 ^st^ Century inspired innovation in pedagogy, and many U.S. schools incorporated ‘active’ and ‘engaged’ learning strategies for the large classroom setting. Parallel to undergraduate medical and health science education, Science, Technology, Engineering, and Math (STEM) undergraduate education transformed, culminating with the Freeman et al ( Freeman *et al.*, 2014) paper and commentary by Wieman ( Wieman, 2014) in Proceedings of the National Academy of Science that debunked the myth that lecture-based teaching has pedagogical value compared to ‘active’ learning strategies.

A critical contextual phenomenon - generation change - has also occurred over the past decade, transitioning from Millennial to Generation Z (born 1995-2010). As of 2018, most learners in the undergraduate phase of post-secondary education are in Generation Z which has implications for teaching and learning in medical and health science education. ( Seemiller and Grace, 2017) We must consider this new generation’s attitudes towards and behaviors with learning in our design of curricula, courses, and pedagogies.

The science of learning blends findings from neuroscience, cognitive, and motivational psychology to inform educators and learners on what works and does not to enhance learning. The publication of *Make it Stick* in 2014 ( Brown, Roediger and McDaniel, 2014), written by established learning scientists, inspired learners to re-think how to get the most from ‘studying’ outside and inside the classroom. It has also challenged educators to develop their courses and curricula in ways that complement how learners learn best.

## TIP 1

Generation Z learners are those born 1995-2010. As Seemiller and Grace ( Seemiller and Grace, 2017) note in their seminal introduction to this cohort: “to..educate, and graduate this new generational cohort effectively, educators must understand the overarching characteristics, perspectives, and styles of these students.” Keep in mind:

• They grew up with smartphones, broadband access, and information is a ‘click away.’
• They prefer ‘hands-on’ learning that can be applied immediately to real life and “..to be actively doing the learning to obtain the most information.” ( *national survey - News @ Northeastern*, 2014)
• They prefer to learn through video content over reading and, as yet, it is unknown if this will equal reading and its ability to develop critical thinking.
• They prefer intrapersonal learning, but they become engaged with collaborative learning when it requires them to use what they have learned on their own.
• Gen Zers prefer in-person instruction and interaction even though they are very adept at using technology. ( LEVIT, 2015)

## TIP 2

Z generation generally learn best by doing and less by listening and are highly motivated to learn so that they can be successful on ‘high stakes’ qualifying examinations for licensure or certification and in the clinical practice setting either as student or employee. The instructor must be knowledgeable about what is expected for these exams and incorporate this into course or curriculum design. Research on motivation in medical students demonstrates that autonomous motivation is enhanced by perceived meaningfulness and value of a course. ( Sobral, 2004), therefore, it’s vital to inform them of this and that the other skills that are used and developed in the ELC include communication, teamwork, leadership (defined and/or shared), and personal accountability - all critical for becoming a health care professional.

A top concern of students about the ELC is that their time both in and out of class is well-spent on what they need to be learning to be successful in the course and beyond. The course objectives should provide this sense of direction and purpose and all assignments and activities need to align with these.

## TIP 3

All health professions educational programs have more content to be mastered than curricular time while Z Gen are career driven and some already have their own business and startups. They use their time more efficiently than previous generations. Faculty must monitor and adjust course expectations with a focus on the information, knowledge, and skill sets that are most vital for success on the course summative and licensure/certification exams, as well the non-cognitive and psychomotor skills inherent with the education of health care professionals.

On the other hand, health professionals must stay current with the literature in their field and this requires facility with acquiring and integrating new findings and information continuously, most often through reading. Therefore, setting appropriately high expectations for deep learning outside of class is paramount for the development of the future practitioner. So, the instructor must determine the reasonable amount and kind of preparation for class time, factoring in the other academic obligations of the learner (competing classes), key concepts and information within the content domain, and an appropriate level of intellectual and professional skills challenge.

Assigning textbook and/or scholarly articles is definitely an expectation. However, the amount of reading students can master and understand varies greatly and depends on the complexity of the reading material. No two sources are alike and six pages in one source does not equal six pages in another. We must choose our sources wisely. Requiring learners to watch several hours of video-recorded lectures must factor in the speed setting that learners generally use - 2-3X! In our experience, we have found that for every hour of in-class learning activity one can expect about 3 hours of out-of-class dedicated effort.

## TIP 4

Learners appreciate clarity on what the course goals are and how they are going to reach them, step-by-step. Using Grant Wiggins and Jay McTighe’s ( Wiggins, G. and McTighe, 2011) approach, one lays out the Desired Results, the Evidence needed to assure achievement of the Desired Results, and the Learning Plan, that details the weekly ‘steps,’ and resources needed along the way, and the depth to which they must be mastered.

Dee Fink has provided a practical and schematic approach (the ‘castle-top’) ( Fink, 2013) for course design so that learning objectives can be met. Complementing the ‘backward design’ paradigm ( Wiggins, G. and McTighe, 2011), one can lay out graphically for the learners what the learning expectations and activities are for each week - both out-of-class and in-class.

**Figure 1.**
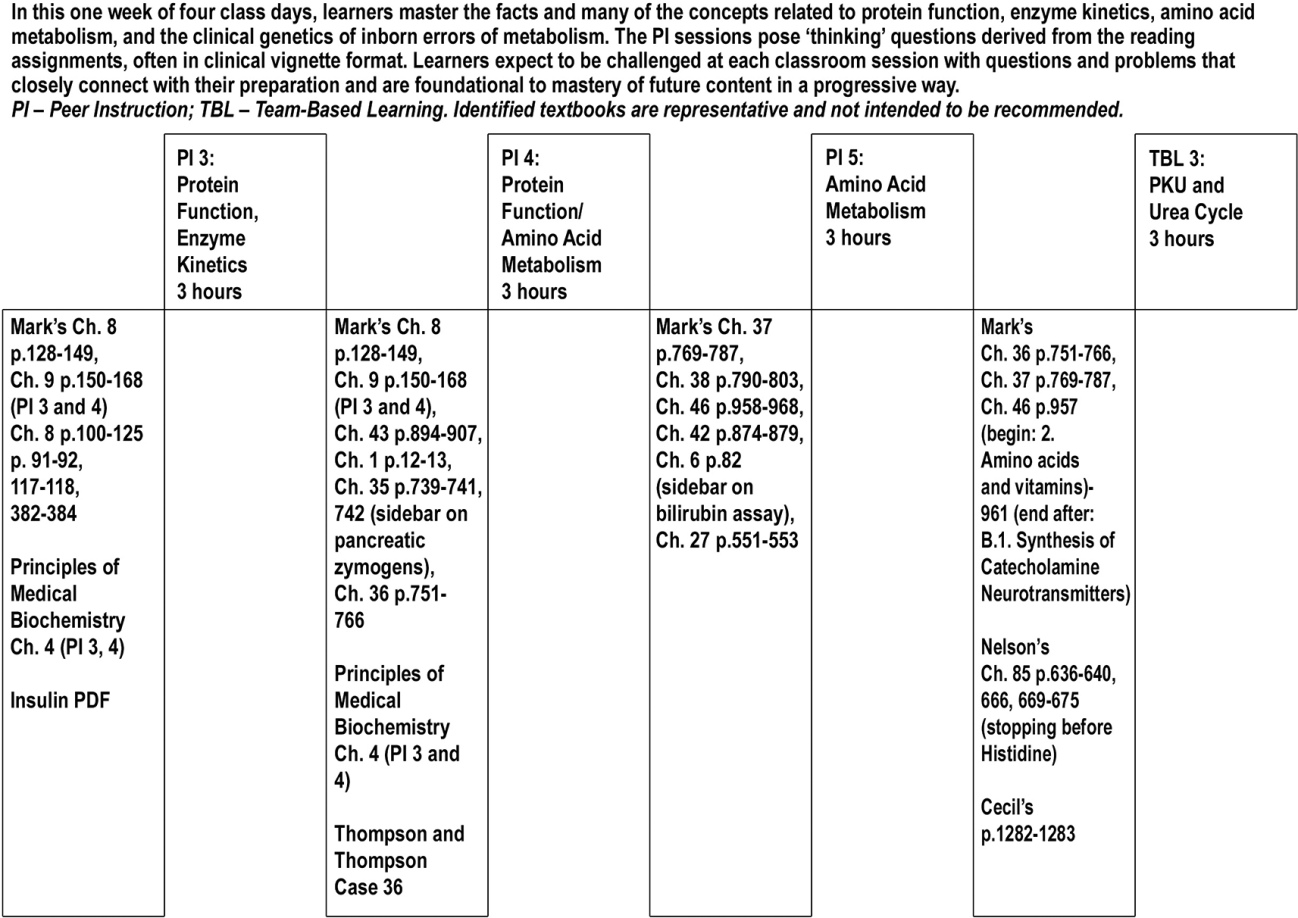
A representative week using the ‘castle-top’ course design model.

Figure 1 shows a week in a medical biochemistry module: there is a theme for the week culminating in a credited but not graded out-of-class on-line ‘quiz’ with questions on key learning points from the week. Each day, including the first day of the week, has an out-of-class assignment that is preparatory for the three in-class Peer Instruction sessions and one Team-Based Learning session with a complex clinical case to solve.

There is often ‘push back’ from today’s learners that there is little or no ‘downtime’ in an ELC - everyday has an assignment often with a formative assessment. They miss the traditional format of lecture/lecture/lecture for three or four weeks, then an exam, for which they only study the week prior! Then after the exam, they can rest for a couple of weeks! Best to clarify early and periodically during the course that achieving the objectives of the course and the program requires a level of discipline and commitment that will pay off and the learning will ‘stick.’

## TIP 5

Apparently, Z generation students prefer to be active and aren’t interested in showing up to sit in a class and just listen and take notes. Make your first class in the course one that is an ELC. If there is not the option of having the registrants prepare ahead for the class, then have something ‘simple’ for them to read/watch as soon as they are seated, e.g. the syllabus or other guide to the course. Engage them in that material with an ELC strategy, highlighting how close reading of the ‘out-of-class’ material makes the ELC activity richer. Ideally, every learner leaves the session knowing where the course is going, the expectations for preparation and achievement, and how to address challenges as they arise. Keep in mind that Gen Z learners prefer in-person over on-line interaction ( Levit, 2015) and on the first day, learners want to get a ‘feel’ for the instructor and the course. A good launch enables easier maintenance and a good landing.

## TIP 6

Gen Z students can quickly decline assignments that they perceive as mundane, overtaxing or dull. A learning contract ( Balan, Clark and Restall, 2015) from the beginning emphasizes the learner’s responsibilities for the learning, much of which will occur outside of the classroom. It can also lead to an early discussion of the responsibilities of the instructor since so many learners want the instructor to tell them all they need to know during classroom time. This does not have to become a cumbersome process for either the learners or the instructor. Either during the first class, conducted in an ELC format, or as an on-line query after the first class or two, ask learners respond to the questions like these:

• What two things will you do to learn in this class?
• Name one thing you will do that will support your peers’ learning.
• What two things do you expect the instructor to do that will enhance your learning?

Gather, categorize, and post for all students so that each student is prompted to reflect on how they responded and whether or not their responses are ‘in-line’ with others in the class. Some responses to the question about the instructor’s role can include “Make sure I get a high score on the board exam,” or “Help condense things down to what’s really important.” This provides the instructor a great opportunity to state what he/she can or cannot do and what is clearly the learner’s responsibility.

Mid-way in the course, use a similar, straightforward format so the whole class can discover what is working and what needs improvement. Keep it transparent. Suggested questions are:

On a scale of 1----to -----10, what is your effort level in preparing for class sessions?

On a scale of 1---to ---10, how well are you making connections between your out-of-class learning and what we do in- class?

What single thing can change about how you are studying that will help you achieve your goal in this course?

As above, gathering this information and sharing it with the whole class is a great way to reinforce the importance of a learning contract and the building of self-reflective capacity for learning.

## TIP 7

For learners who are unacquainted with the ELC and its requirement for considerable out-of-class effort, it can be quite a challenge to transition to a self-directed learning attitude. For the instructor, one must carefully consider what kind and level of ‘guidance’ learners in the health professions need. The two resources we present may be helpful for communicating an ‘approach’ to the learning, not to make the learning easier.

### Study Guide

Study Guides that are specific to each of the ELC sessions are used by learners to focus on what in the content assignment is most important. Here are three steps for creating a study guide ( Hollingsead *et al.,* 2004)

(1) Laying out the key concepts, either as objectives for the session or on a graphic form that taps the landscape of the assignment is a good start.

(2) Identify the 2-3 areas that are the most challenging to learn and suggest an approach. For example, if the session is an introduction to neoplasia: the two biggest challenges can be mastering the vocabulary (“be able to define all unfamiliar words and terms in the reading;”) linking core molecular genetics to pathological mechanisms (“carefully review the major take-aways from last week’s sessions on molecular genetics first.”)

(3) Suggest a strategy to master the amount of reading without re-reading or highlighting, e.g. “After each of the subsections, pause and create from memory a concept map that formalizes the key words and concepts you just studied.”

Or, “At the end of each section, do 5 MCQs from the question bank on neoplasia, graph your progress at the end of the chapter.”

Since Generation Z gravitates to videos, identify one or two video presentations from the web that you feel provides a good overview, but stress the importance of reading as the only way to really get a handle on neoplasia or any complex topic.

Study guides should never make the learner feel that the workload of the assignment is reduced by the study guide. It should give focus for the effort required. The study guide should stimulate the thinking about the material, and as the course progresses, substitute specific guidance with assignments/questions that emphasize developing reflective capacity: “After reading and studying this chapter, write out the three concepts that were most difficult to understand and remember.”

### Framing" video presentations

Learners definitely like to see and hear their instructors. Creating a ‘Framing’ video presentation can be an effective way to engage them in tackling out-of-class assignments. These are best when they are brief and focused - no more than 15 minutes and with clear goals such as “In this video, I’m going to show you how I approach describing the morphology of histologic images. I’ll be using some new terms, which are listed in the study guide for you to know for class. I will be demonstrating my thought process when I look at a slide, and at the end of this short video, you should be able to practice my approach with the materials in the textbook and lab.”

## TIP 8

• Videos of lectures, multi-media, interactive technology-supported formats

When audio-visual recording technology became available to record live lectures so that learners really didn’t have to come to class, attendance at lectures became spotty at best, and learners would speed up the video presentations so that a 50-minute lecture could be completed in 15 minutes. Ironically, human attention span for a lecture hovers around 15 minutes anyway. The ‘flipped-classroom’ became characterized as learners listening to AV lectures as preparation for the in-class learning activities. Reading became even more optional, which has suited Gen Z. Health professions learners now have a huge selection of video lecture options on the internet, some free and some through one or another ‘board prep’ proprietary services. The quality of these varies, but learners usually find those that they feel prepares them for their assignments and exams.

There is now a whole field devoted to multi-media learning and Mayer ( Mayer, 2008) has highlighted that learners best learn with a combination of animation with narration and text, and there are many computer-assisted programs and software that make creating engaging audio-visual presentation easier for the content-expert. Creating multi-media learning tools that truly supplement and not supplant close reading is an increasingly popular option, and there are good guidance resources for doing this. Indeed, well-done multi-media instruction does increase student engagement and learning, though it is still undetermined if critical thinking is developed without reading. ( Carmichael, Reid and Karpicke, 2018)

• Reading textbooks and other publications:

Gen Z resists reading, and many well-established biomedical science text books come with accompanying access to audio and or video lectures on the key topics. Learners prefer to start with the AV coverage of assigned material and then may delve into the related reading. The jury is ‘out’ on whether or not the kind of critical thinking that we expect health professionals to have develops without extensive reading. ( Carmichael, Reid and Karpicke, 2018) In any case, we recommend for preparation for in-class activities and the course summative exams, and we must/should specify the depth to which the reader must attend.

Heiner et al ( Heiner, Banet and Wieman, 2014) found that the most effective way to get students to read pre-class assignments by attaching an on-line ‘quiz’ following a reading assignment, one where the questions were answerable through careful reading and related directly to the next class learning activities. TBL incorporates a quiz/test on the out-of-class assignment as an integral component of the strategy to incentivize students to prepare and engage them with peers early in a session.

• Lab or Field-work assignments

More commonly, science courses with laboratory require students to prepare for the laboratory time through reading or watching some instructional video material. But, the laboratory learning can be further enhanced when it is deemed preparatory to the in-class learning activities

## TIP 9

Consider the ELC as a continuum of learning where the division between in-class and out-of-class activities should be experienced as minimal by the learner and instructor. To foster this from day one of class, one can pose questions, either live or through the learning management system such as: In no more than 10 words:

• “What went well for your learning this week?”
• “What helped you connect the heavy out-of-class reading this week to the problem-sets in class?”
• “What will you do differently next week?”

*Make It Stick* ( Brown, Roediger and McDaniel, 2014) has many examples of learners who developed life-long-learning habits of retrieval, generation, and reflection and provides the evidence on the effectiveness of these metacognition/reflective practices for improving learning. Since health professions education requires the development of non-cognitive competencies such as interpersonal, communication, and team-work, and these skills are integral to learner success in any ELC, posing questions such as:

• This past week: what is the one thing you did to support your group’s learning?
• What one behavior would you like your peers to do to help your learning?
• This coming week, name one thing you will do differently to enhance your group’s learning?

These are representative questions that stimulate the learners to consider their roles and responsibilities for their own and their peer group’s learning. In collaborative learning where small group leadership shifts through a process of distributed leadership, providing prompts to pay attention to one’s inner dynamics and the learning group keeps a heightened awareness of the collaborative process so vital to learning both in and out of the classroom.

Since all ELC courses require some form of collaborative learning, i.e. permanent learning teams (TBL) or smaller clustering of learners that changes from session to session (PI), posing reflective practice questions periodically about “How are you contributing to your peers learning both in the classroom and outside?”

Teaching between learners outside of class can mirror what is happening in the classroom via tools such as GoogleDocs, chat rooms, discussion boards, Zoom, Skype. It’s never good to require students to ‘get together’ outside of class to study or complete a project but including reflective practice queries about how they are supporting one another’s learning will enhance their engagement with course material and can build greater awareness of their roles of leadership (distributed and/or designated) within their peer group. ( Alizadeh *et al.*, 2017)

## TIP 10

Since the amount of time that learners need to spend out-of-class to be successful in an ELC is much more than the time in the classroom, another tool that Generation Z learners use a great deal is social media, such as Twitter.

As the 2011 publication of Junco et al ( Junco, Heiberger and Loken, 2011) demonstrated, the instructor can easily expand discussion started in the classroom by asking a probing question, ask a probing question about the assignment prior to the release of the pre-class quiz; learners can network for study groups, share documents, share tips on preparation for an upcoming classroom session or exam.

## TIP 11

Using technology support available for the ELC (on-line quizzes, tests, ARS clickers, IF-AT e-forms, social media, discussion board, on-line chat), one can easily monitor both an individual student’s achievement as well as the class as a whole. Even the first couple of classes will provide data (pre-class on-line quizzes, reflective comments, questions; in-class performance on questions; post-class quizzes, reflective comments) that can identify learners who are struggling to master the out-of-class work, enabling you to ask them questions about their study approach and counsel them. Often, the whole class struggles initially with the volume and depth of the required assignment and needs encouragement to ‘stick with it.’ As *Make It Stick* wisely notes for the learner the greatest learning often occurs when one is challenged in unexpected ways. ( Brown, Roediger and McDaniel, 2014, pp 67-101) The ELC provides the instructor with early and useful information on learner progress with early opportunities to adjust the course and/or assist individual students in ways never possible in traditional lecture format.

Another valuable aspect of the ELC is the frequent feedback for the learner and also for the instructor. Using ‘Learning Analytics,’ available and supported by technology, provides the learner with daily data on ‘Did I learn this well?’ ‘Here’s where I still need more work.’ As an example, having a short 5 question ‘quiz’ on-line prior to the classroom component drawn from the assignment, when tracked across the course, will provide the learner with marks of progress for how to learn. Similarly, the instructor can track individuals and the class as a whole on the daily out-of-class achievement. Combining this with the in-class learning activities’ data provides both learner and instructor with robust feedback for adjusting both study approaches (learner) and level of difficulty (instructor).

All instructors become concerned for students who struggle early on in a course. Sometimes, the frequent feedback discourages them because they don’t feel they can ‘get ahead.’ This, predictably, leads to a downward spiral of moral. With the ELC strategy, the instructor (or academic advisor) can tap into the data emerging from the first week and communicate with any student in this category and explore his/her learning approach and study habits, level of reflective capacity, e.g. “I think it’s because I hate to read,” or some impinging psychosocial factor in personal life that may need professional intervention.

## TIP 12

Your institution or program will use some standard format for eliciting feedback from the students on your course design and teaching. You have the option, particularly towards the conclusion of the course, to gather additional information, including how well the out-of-class assignments enhanced the learning. Attached is a sample set of queries that capture both useful quantitative and qualitative data.

• As the course progressed, to what degree did the out-of-class assignments prepare you for the in-class activities?

(very little) 0-------------------------------------------------------------------------10 (very much)

• To what degree did they help you achieve your goals for the summative exams?

0-------------------------------------------------------------------------------------10

• In addition to the REQUIRED assignments, name two other resources that you found helpful.
• If you were starting the course today, what one thing (looking back!) would you have liked to have known about how to meet your goals for the course?

## Conclusions

Health professions education is changing rapidly to incorporate teaching and learning activities that are grounded in the science of learning and fit with the learning preferences of the Generation Z learners. The ELC, often called the ‘flipped-classroom,’ is beginning to dominate the pedagogical strategies selected by instructors. We have crafted this set of ‘Tips’ to assist instructors on how to design the ‘out-of-class’ learning activities so that they link well with and enhance the ‘in-class’ learning.

## Take Home Messages

• Instructors using one of the Engaged Learning Classroom (ELC) strategies must attend to how learners can best learn the content for the in-classroom learning activities.
• Generation Z learners like the face-to-face learning that can occur in the ELC as long as it is focused on what they need for summative assessments. They also want the out-of-class preparation assignments to link tightly to the in-class activities and therefore the summative assessments.
• There is evidence that well-designed and delivered multi-media instruction is effective for learning, however, it is unknown if it develops critical thinking as does reading

## Notes On Contributors

Maryam Alizade, PhD, is Director of the Faculty Development Unit, Education Development Center (EDC) with the Education Development Office at the School of Medicine of Tehran University of Medical Sciences. Her PhD is in Medical Education.

Irina Overman, M.D. is Director of Foundations of Clinical Practice and Associate Clerkship Director in Internal Medicine at the Wright State University Boonshoft School of Medicine. She is both an internist and geriatrician.

Mohamad AlJasem is a medical student at School of Medicine International Campus, Tehran University of Medical Sciences, Tehran, Iran. He is interested in the field of medical education.

Dean Parmelee, M.D. is Director of Educational Scholarship and Program Development at the Wright State University Boonshoft School of Medicine. He has played a major role in helping transform his school’s curriculum to be ‘lecture-free.’

## Declarations

The author has declared that there are no conflicts of interest.

## Ethics Statement

No human subjects or data from humans collected.

## External Funding

This article has not had any External Funding
