# Supplementary material for: Preparing Learners for Learning in the Engaged Learning Classroom
Source: MedEdPublish (2016). 2019 May 31;8:120. [Version 1] doi: 10.15694/mep.2019.000120.1 (PMC10712513; doi:10.15694/mep.2019.000120.1)
Supplement: Peer review history

# Reviewer report: Reviewer response for version 1

**DOI:** 10.21956/mep.19850.r29948

**Published:** 2019-08-02

**Authors:** Barbara Jennings

**Affiliations:** 1 Norwich Medical School

This review has been migrated. The reviewer awarded 4 stars out of 5 There is a perfect storm for medical educators right now - caused in part by a rapid increase in the number of learners, with insufficient faculty to teach all of them face to face (nor indeed actual classroom space in which to do this). This is compounded by a change in expectation from the new generation of learners, who have been brought up with access to almost limitless information and who have developed the skills to access it efficiently.I think that this excellent article, about design and use of “out of class” activities, offers us all some solutions to consider. The authors have also provided the readers with an overview of the science and educational theory that underpins their tips. I really enjoyed reading this article and will be sending it to all module teams that I work with, and taking on board many of the suggestions for my own flipped classrooms and online teaching delivery. Thank you.

Reviewer Expertise:

NA

No decision status is available

# Reviewer report: Reviewer response for version 1

**DOI:** 10.21956/mep.19850.r29950

**Published:** 2019-06-04

**Authors:** Subha Ramani

**Affiliations:** 1 Harvard Medical School, Brigham and Women's Hospital

This review has been migrated. The reviewer awarded 4 stars out of 5 The authors have written a set of Tips to foster an Engaged Learning Classroom (ELC). They use principles from science of learning to anchor their tips. They describe a variety of established strategies to establish student engaged learning such as Team-Based Learning (TBL), Peer Instruction (PI), Case-Based Learning (CBL) etc. The paper is well written and well referenced. The tips flow well and are written from the teacher and learner perspectives. They have clearly outlined the before, during and after phases. Finally, they have alluded to alignment between learning objectives, content and assessment.I have one comment about the complete focus on Gen Z students as I am not sure stated principles from cognitive neuroscience and educational theories apply just to this generation. Almost all the tips for engagement of learners are not generation specific. In fact, most references including backward design and 'make it stick' do not refer to a certain generation of learners. I would have liked to see a mention that many principles are relevant to all learners and what might be different about Gen Z learners.

Reviewer Expertise:

NA

No decision status is available

# Reviewer report: Reviewer response for version 1

**DOI:** 10.21956/mep.19850.r29949

**Published:** 2019-05-31

**Authors:** Lee HangFu

**Affiliations:** 1 Windsor University School of Medicine

This review has been migrated. The reviewer awarded 4 stars out of 5 A nice paper for the Gen Z learners. Perhaps the author(s) can reference more on the personality, characteristics and learning habits of the Gen Z. Overall a nice paper and easy to read.

Reviewer Expertise:

NA

No decision status is available

# Reviewer report: Reviewer response for version 1

**DOI:** 10.21956/mep.19850.r29951

**Published:** 2019-05-31

**Authors:** Felix Silwimba

**Affiliations:** 1 University of Lusaka

This review has been migrated. The reviewer awarded 5 stars out of 5 This paper in detail and depth describes the approaches to educating the generation Z learners, who are as well described as visual learners. I have picked several points and ideas to apply in my development as an educator. I recommend it for all medical educators even those in LMIC like where I’m based

Reviewer Expertise:

NA

No decision status is available
